# The global prevalence of eating disorders in children and young people: a systematic review and meta-analysis

**DOI:** 10.1007/s00787-025-02933-0

**Published:** 2026-01-27

**Authors:** Clara Faria, Kian Daneshi, Aslihan Baser, Henrike Mauersberger, Abigail G-Medhin, Emma Soneson, Simon White, Joanna Anderson, Tamsin Ford

**Affiliations:** 1 Department of Psychiatry, University of Cambridge, Cambridge, UK; 2 School of Medicine, University of Sheffield, Sheffield, UK; 3 GKT School of Medical Education, Kings College London, London, UK; 4 Department of Psychiatry, University of Oxford, Oxford, UK

**Keywords:** Eating disorders, CYP, Children and young people

## Abstract

The increase in eating disorder (ED) presentations among children and young people (CYP) during the Covid-19 pandemic represents a global health concern, given the associated morbidity and mortality associated with these conditions. We conducted a meta-analysis to estimate the worldwide pooled prevalence of EDs in CYP at a population level. We searched MEDLINE, EMBASE, PsycINFO and LILACS for all English-language studies reporting population level ED prevalence data between January 2013 to February 27th, 2024. The primary outcome was overall prevalence of EDs. We used a random-effects model for the meta-analysis, I^2^ statistics to assess heterogeneity, and the Hoy scale for quality assessment. This study is registered with PROSPERO, number CRD42022333223. Sixteen studies including 77,714 children and young people from 12 countries met eligibility criteria and were included in the systematic review, whilst twelve studies with 56,758 participants provided data suitable for inclusion in the meta-analysis. Study quality was moderate; ten studies were classified with a low risk of bias, one with moderate and five with high. The global point prevalence for any ED was 5.23% (95% confidence interval (CI) 0.41 to 10.05, I^2^= 99.95%). The commonest type was Other Specified Feeding or ED (point prevalence of 4.88%, 95% CI 1.46 to 8.30, I^2^= 99.42%), which, like all types of ED was more common among girls (5.25%, 95% CI 0.32 to 10.18, I^2^= 99.69%) than boys (3.97%, 95% CI 0.45 to 7.49, I^2^= 97.77%), although confidence intervals overlapped. Our findings illustrate the urgent need to expand service provision as well as to evaluate and develop strategies for early ED identification in children and young people, given a global prevalence of 1 in 20.

**Supplementary Information:**

The online version contains supplementary material available at 10.1007/s00787-025-02933-0.

## Key points and relevance

Children and young people (CYP) are disproportionately affected by eating disorders (EDs), which tend to develop in adolescence.

There has been an increase in clinical presentations of EDs among CYP in high income countries

EDs in CYP are common, with a global point prevalence of approximately 1 in every 20. Our subgroup analyses showed that Other Specified Feeding or eating disorder (OSFED) is the most common type of ED among CYP.

Service provision needs to increase to support CYP with EDs, which are much commoner in this age group than previous studies suggested.

Healthcare workforce training is another key area of attention. General practitioners and primary care professionals need to be aware of ED symptoms to ensure early identification and timely referral to specialist services when needed.

**Supplementary Information:**

The online version contains supplementary material available at 10.1007/s00787-025-02933-0.

## Introduction

 Eating disorders (EDs) are currently a major global public health emergency among children and young people (CYP). Evidence suggests that the Covid-19 pandemic significantly undermined mental health, particularly for young people, both in low- and middle-income countries as well as in high income countries [1, 2]. Specifically, there has been an increase in clinical presentations of EDs among CYP in high income countries [3, 4]. In the United Kingdom, a cohort study examining all hospital admissions for children and young people over the past decade found a 514% increase in admissions related to eating disorders [5].

 EDs are defined by disturbed perceptions and behaviours towards weight, body shape and/or eating [6].There are six main types of EDs described in the Diagnostic and Statistical Manual of Mental Disorders (Fifth Edition) (DSM-5): anorexia nervosa, bulimia nervosa, binge-eating disorder (BED), Avoidant and Restrictive Food Intake Disorder (ARFID), pica and rumination disorder [7, 8]. Additionally, the DSM-5 includes two residual diagnostic classifications: 'other specified' (OSFED) and'unspecified' (UFED) feeding and eating disorders. All EDs can cause severe impairment in physical health and psychosocial functioning [6]. Indeed, data shows that individuals diagnosed with anorexia nervosa are five to eight times more likely to die in the first ten years following the diagnosis when compared to individuals in the general population [9, 10].

 We cannot be sure if the increase in ED clinical presentations reflects a true increase in population-level prevalence or increased help-seeking behaviours during the pandemic and in its aftermath. Particularly in the initial stages of an ED, CYP may conceal their difficulties, for some due to the ego-syntonic nature of the symptoms and for others due to shame at the behaviours involved [6]. The situation during lockdowns where families spent increased time together may have allowed parents better insight into what their children were eating, but equally, the loss of structure, control and opportunities to exercise and to undertake hobbies may have pushed more vulnerable adolescents into clinical level difficulties [11].

 EDs should not be confused with disordered eating, which comprise subclinical difficulties that are extremely common among CYP. Disordered eating can be understood as a set of dysfunctional eating behaviours and beliefs that lead to an increased likelihood of broader difficulties with eating but are not clinically impairing and do not meet diagnostic criteria for an ED [2, 12]. The aetiology of eating disorders in children and young people is complex and involves the interplay of many genetic, behavioural, and environmental factors [6, 13], and includes disordered eating [14, 15]. A recent meta-analysis reported that 22.4% of CYP worldwide suffer with disordered eating [12].While disordered eating is a potential indicator of vulnerability to ED, it does not reflect level of clinical need for specialist ED services. Similarly, 12.3% of 11- to 16-year-olds and 59.4% of 17- to 19-year-olds participating in the national survey programme providing official government statistics to the English Department of Health and Social Care estimated reported eating difficulties [2]. This same survey estimated that 2.6% of 11- to 16-year-olds and 12.5% of 17- to 19-year-olds in England were living with an ED compared with 0.5% and 0.8%, respectively, in 2017, which suggests a true increase in prevalence. However, it is important to highlight that it is a single study of a survey sample that did not intend to be a longitudinal study. It remains unclear if the same increase in ED prevalence in CYP was also observed in other countries, or how accurately the findings reflected changes in England.

 Accurate population prevalence estimates are essential to predict service need and to determine whether increased early intervention strategies at primary care and at school level are necessary. A recent systematic review and meta-analysis on the CYP healthcare use for eating disorders during the COVID-19 pandemic showed paediatric utilisation of ED services increased substantially, particularly among girls and adolescents [16]. In the UK context specifically, the number of referrals to the child and adolescent eating disorders services almost doubled in the aftermath of the covid-19 pandemic [17], with services struggling to manage waiting lists. While the surge in mental health services use is not exclusive of eating disorders [16], it is particularly important for improved treatment outcomes that patients suffering from EDs can readily access treatment [6].

Therefore, we conducted a systematic review and meta-analysis to estimate the worldwide pooled prevalence of EDs from 2013 (the introduction of DSM-5) and whether this has changed over time. To the best of our knowledge, this is the first systematic review and meta-analysis to estimate the worldwide prevalence of clinically impairing EDs in CYP. 

##  Methods

### Search and inclusion criteria

We followed an *a priori* protocol registered in PROSPERO (CRD42022333223) and the Preferred Reporting Items for Systematic Reviews and Meta-analyses (PRISMA) guidelines [18].We searched four databases (MEDLINE, EMBASE, PsycINFO and LILACS) from January 2013 (year of publication of DSM-5) to June 2022 and then updated the search. All the searches were updated using the same strings on 27th February 2024. The following search keywords were used in Ovid, with equivalent syntax for the other repositories and databases: (1) adolescent* [tw] OR teen* [tw] OR child* [tw] OR youth [tw] OR “young person” [tw] and (2) “Feeding and eating disorders” [tw] OR “Atypical anorexia” [tw] OR Anorexia [tw] OR “Anorexia nervosa” [tw] OR ARFID [tw] OR Bulimia [tw] OR “Binge-eating disorder”. For the complete search strategies, see Supporting Material S1. We also checked the reference lists of the included studies and used forward citation searching to identify any additional potentially relevant papers. Search results were managed using CADIMA (https://www.cadima.info/index.php).

 Inclusion criteria were (1) participants: CYP up to the age of 24 years from community-based samples; (2) outcome: the prevalence of EDs in CYP using DSM-5 or ICD-10 or −11 diagnostic criteria; (3) study design: population surveys, cross-sectional studies and cohort studies if they reported data on point prevalence of EDs; (4) sampling strategy: studies that used a clearly defined sampling frame. While no restrictions were placed on the country of the study, studies had to at least have an abstract published in English. Studies that included adults over the age of 24 years could be included provided that data on CYP were reported separately. Exclusion criteria included convenience samples, studies published before 2013 and studies which did not have at least an abstract in English. Two studies did not report data stratified by age group; we contacted study authors to request the data for this meta-analysis and received no response. We, therefore, did not include those studies. Four studies reported lifetime prevalence only. We also did not include these studies in the meta-analysis as lifetime prevalence and point prevalence measure different constructs.

 CF screened all the titles and abstracts. KD, FA, HM, AB and AG double screened 20% of the titles and abstracts in total. Full texts of the remaining studies identified in the first stage were then screened against the inclusion and exclusion criteria by CF and 20% of those records in the first stage were also double screened in parallel by KD. Any disagreement was resolved by consensus.

### Data extraction

 Two authors (CF, KD) independently extracted data, with any discrepancies resolved by discussion. We extracted data on study characteristics, sample characteristics, study methods, case definition and prevalence data using a predesigned data extraction form (see Supporting Material S2).

### Quality appraisal

Two authors (CF, KD) independently assessed the quality of the included studies with the Hoy scale adapted for prevalence studies, which is composed of 10 items each scored as 0 (low risk of bias) or 1 (high risk of bias) [19].This scale does not include an overall numeric rating of risk of study bias, but we categorised the overall risk of study bias based on assessment of risk of bias of ten individual items [19]. ^.^Studies rated as having a high risk of bias in three or more items of the scale were classified as having an overall high risk of bias, whilst studies rated as having a high risk of bias in up to one item were classified as having an overall low risk of bias. The complete scale can be found on Supporting Information Table S3. Any discrepancies were resolved by discussion.

### Data synthesis and analysis

 We anticipated considerable between study heterogeneity due to the broad clinical definitions encompassing eating disorders. Therefore, we performed a random-effects meta-analysis to calculate the global prevalence of EDs in CYP with R using the *metafor* pac*k*age. All studies that provided at least one point prevalence estimate were included in the meta-analysis. When possible, we conducted separate meta-analyses to estimate the pooled prevalence of any ED stratified by gender and the pooled prevalences of specific EDs. Studies that provided estimates for one type of ED only (e.g. anorexia nervosa) or gender stratified estimates only but did not provide an overall estimate for any ED were included in the sub-group meta-analyses but not in the any ED meta-analysis [20–22]. The I^2^ statistics was used to assess between-study heterogeneity; significant heterogeneity was indicated by I^2^≥50% [24]. Cochran’s Q test was used to assess whether the observed heterogeneity in prevalence estimates across studies was statistically significant. To explore potential sources of heterogeneity, we undertook *post hoc* meta-regressions using univariate mixed linear regression models and maximum likelihood estimators of geographic location, year of data collection, sample age range, sample representativeness, sampling frame, study design, diagnostic criteria, diagnostic interview, informants and prevalence timeframe. There were insufficient studies reporting prevalence estimates to undertake meta-regressions for the specific ED types.

## Results

### Study selection

**Fig. 1:**
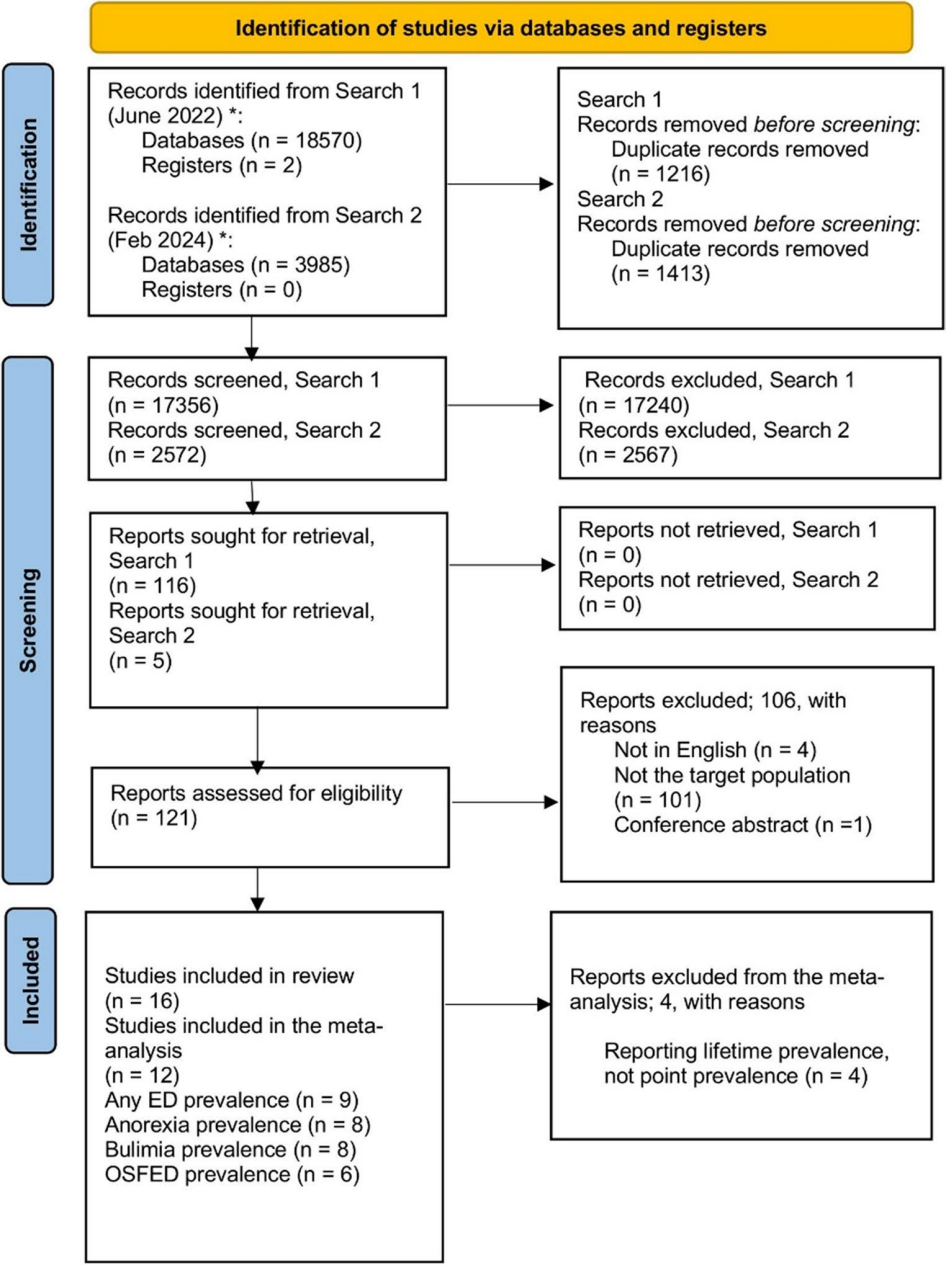
Prisma chart – identification of studies

The initial searches identified 18,572 abstracts (see Fig. 1). After duplicates were removed, 17,356 abstracts were screened, with 116 papers selected for full text review. The interrater reliability was excellent (Cohen’s Kappa for title and abstract screening 0,85).

 An additional five studies were identified through review of the references of all articles examined for inclusion; thus, a total of 121 papers underwent full text review. The updated search (February 2024) identified 2,572 abstracts after duplicates were removed. Five additional papers from the updated search were selected for full text review. Sixteen studies with 77,714 CYP from twelve countries met eligibility criteria after full text screening and were included in the analysis [2, 20–23, 25–35]. Figure 1 describes the identification and screening process based on PRISMA guidelines.

### Study characteristics

 Detailed characteristics and references of the included studies are reported in Table 1. Four studies reported lifetime prevalences and thus were not included in the meta-analysis [20, 23, 34, 35].This left twelve studies that could be included in the final meta-analysis, with data from 56,758 CYP, of whom 25,561 (51.26%) were girls or young women [2, 21–23, 25–33]. Most of the research was conducted in European countries and the United Kingdom (*n* = 7), followed by Iran (*n* = 3), United States (*n* = 2), Burkina Faso (*n* = 1), Canada (*n* = 1), and Australia (*n* = 1). Final sample size ranged from 696 to 27,111 across studies and thirteen studies used some form of complex sampling method such as randomised probabilistic sampling in their prevalence estimates [2, 20–23, 28–32, 35] but only five applied weights to generalise their prevalence estimates to the national population [2, 28, 31, 33, 35].

**Table 1:** Summary of the studies included in the systematic review

Study (year of publication)	Country	Sampling frame	Age range (years)	Prevalence type	Sample size	Diagnostic tool used	Eating disorder type	Informant	Diagnostic criteria	Prevalence estimate (95% CI)	Timeframe of assesment
Alvarez-Male (2013) *	Spain	School	12–20	Point prevalence	1342	EAT + The Eating Disorder Examination Questionnaire	Any eating disorder	child/young person	DSM-5	4.11% (3.05–5.17)	one month
Chen (2019)	Taiwan	School	7–14	Lifetime prevalence and 6-month prevalence	9560	Kiddie Schedule for Affective Disorders and Schizophrenia (KSADS)	ARFID and Anorexia	child/young person + parent	DSM-5	0.5% (0.3–0.7)***** 0.2% (0.0–0.4)*******	Lifetime and 6 months
Ernst (2017) *	Germany	School	13–15	Point prevalence	1654	Structured Inventory for Anorexic and Bulimic Syndromes (SIAB-S)	Any eating disorder	child/young person	DSM-5	1.15% (0.64–1.66)	three months
Flament (2017) *	Canada	School	11–20	Point prevalence	3022	The Eating Disorder Diagnostic Scale (EDDS)	Any eating disorder	child/ young person	DSM-5	3.7% (2.8–4.7)	Three months
Vizard (2018) *	England	Registration with a general practitioner	11–19	Point prevalence	4057	Development and Well-Being Assessment (DAWBA)	Any eating disorder	child/young person + parent	DSM-5 and ICD-10	0.8% (0.2–1.4)	one month
Newlove Delgado (2023) *	England	Registration with a general practitioner	11–19	Point prevalence	1263	Development and Well-Being Assessment (DAWBA)	Any eating disorder	child/young person + parent	DSM-5 and ICD-10	12.5% (8.3–16.7)	one month
Glazer (2019)	United States	Household	9–15	Lifetime prevalence	9031	McKnight Risk Factor Survey (MRFS) + self-designed questionnaire	Bulimia Nervosa, Binge Eating Disorder	child/young person	DSM-5	6.1%*** (5.6–6.6) 2.1%**** (1.8–2.4)	Lifetime
Larsen (2020) *	Czech Republic	Schools	12–17	Point prevalence	4430	The Eating Disorder Diagnostic Scale (EDDS)	Bulimia Nervosa	child/young person	DSM-5	Females 11.4% (10.2–12.6) Males 3.8% (2.9–4.7)	three months
Mitchison (2019) *	Australia	Schools	11–19	Point prevalence	5072	The Eating Disorder Examination Questionnaire	Any eating disorder	child/young person	DSM-5	22.2% (21.06–23.34)	Past one month
Mohammadi (2019) *	Iran	Household	6–18	Point prevalence and lifetime prevalence	27111	Kiddie Schedule for Affective Disorders and Schizophrenia (K-SADS-PL)	Any eating disorder	child/young person + parent	DSM-5	0.89% (0.81–1.10)	Past 0.5 month
Rauof (2015) *	Iran	Household	3–18	Point prevalence	1990	EAT – 26, Structured Clinical Interview for DSM Disorders (SCID)	Any eating disorder	child/young person	DSM-5	0.25% (0.03–0.47)	Past three months
Rozzell (2019) *	United States	Multiple sampling frames	9–10	Point prevalence	4524	Kiddie Schedule for Affective Disorders and Schizophrenia (KSADS)	Any eating disorder	child/young person	DSM-5	1.4% (1.0–1.8)	Past 0.5 month
Silen (2020)	Finland	Household	21–26	Lifetime prevalence	1347	Structured Clinical Interview for DSM Disorders (SCID)	Any eating disorder	child/young person	DSM-5	10.5% (8.9–12.4)	Lifetime
Smink (2014) *, **	Netherlands	Household	9.10	Point prevalence	1597	The Composite International Diagnostic Interview + SCID-I	Any eating disorder	child/young person + parent	DSM-5	Females 3.7% (2.6–5.2) Males 0.5% (0.1–1.4)	Past three months
Terhoeven (2020) *	Burkina Faso	Households	12–20	Point prevalence	696	SCID-5 + The Eating Disorder Examination Questionnaire (EDE-Q)	Anorexia nervosa	child/young person	DSM-5	Females 0.6% (0.03–1.17)	Past one month
Yazdi (2020)	Iran	Household	6–18	Lifetime prevalence	1028	Kiddie Schedule for Affective Disorders and Schizophrenia (KSADS)	Bulimia Nervosa	child/young person	DSM-5	0.1% (0.02–0.6)	Lifetime

*Included in the meta-analysis

**Median age reported, age ranges not available

***Estimate for Binge Eating Disorder

****Estimate for Bulimia Nervosa

***** Estimate for ARFID

****** Estimate for Anorexia

 Most studies used households as sampling frame (*n* = 7), followed by schools (*n* = 6), registration with a general practitioner (*n*= 2), and multiple sampling frames (*n* = 1). There was high variability in the diagnostic tool used to ascertain ED diagnosis within studies. Five studies utilised more than one type of diagnostic interview to ascertain diagnosis. The most used diagnostic tool was the Structured Clinical Interview for DSM Disorders (*n*= 3) and Eating Disorder Examination Questionnaire (*n*= 3), followed by the Kiddie Schedule for Affective Disorders and Schizophrenia (*n*= 3), the Development and Well-Being Assessment Interview (*n*= 2) and the Eating Disorder Diagnostic scale (*n*= 2).

 Point prevalence estimates in the included meta-analysis studies were heterogeneous and ranged from 0.25% to 22.2% for any ED. The studies estimating lifetime prevalence, which were not included in the meta-analysis, also provided varying figures. Two studies analysed the community lifetime prevalence of bulimia. Glazer et al.’s US-based study provided an estimate of 2.10% while Yazdi et al.’s Iranian-based survey estimated it around 0.10%. On the other hand, Silen et al.’s Finnish study estimated the lifetime prevalence of any ED around 10.5%. Chen et al estimated the lifetime prevalence of ARFID and anorexia to be 0.5% and 0.2%, respectively.

### Risk of bias

**Fig. 2:**
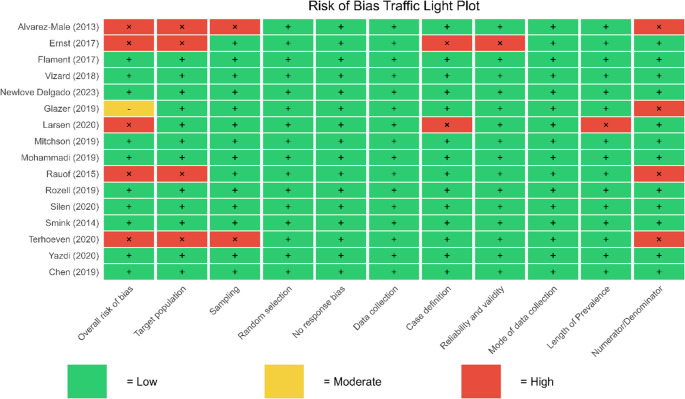
Risk of bias classification

 Two assessors rated each study as low, high, or moderate risk of bias, demonstrating substantial inter-rater agreement (Cohen's *κ* = 0.72). Ten studies were classified with a low risk of bias [2, 21, 23, 26, 28, 30, 31, 33, 35] one with a moderate risk of bias[20] and five [22, 25, 27, 29, 32] with a high risk of bias (see Figure 2 for more detail).

### Meta-analyses

**Fig. 3:**
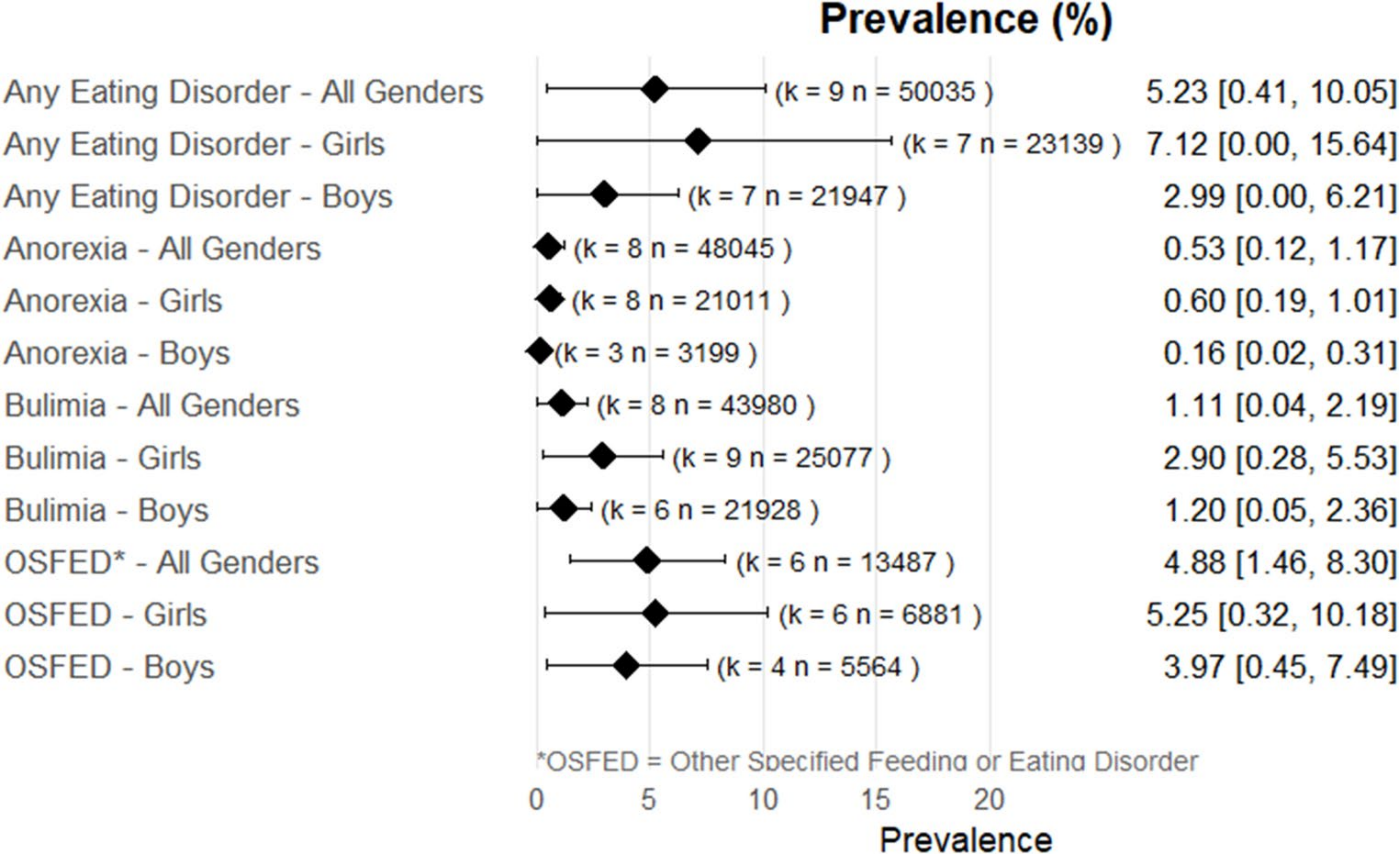
Summary Forest plot of the prevalence of any eating disorders and specific eating disorders (%)

**Fig. 4:**
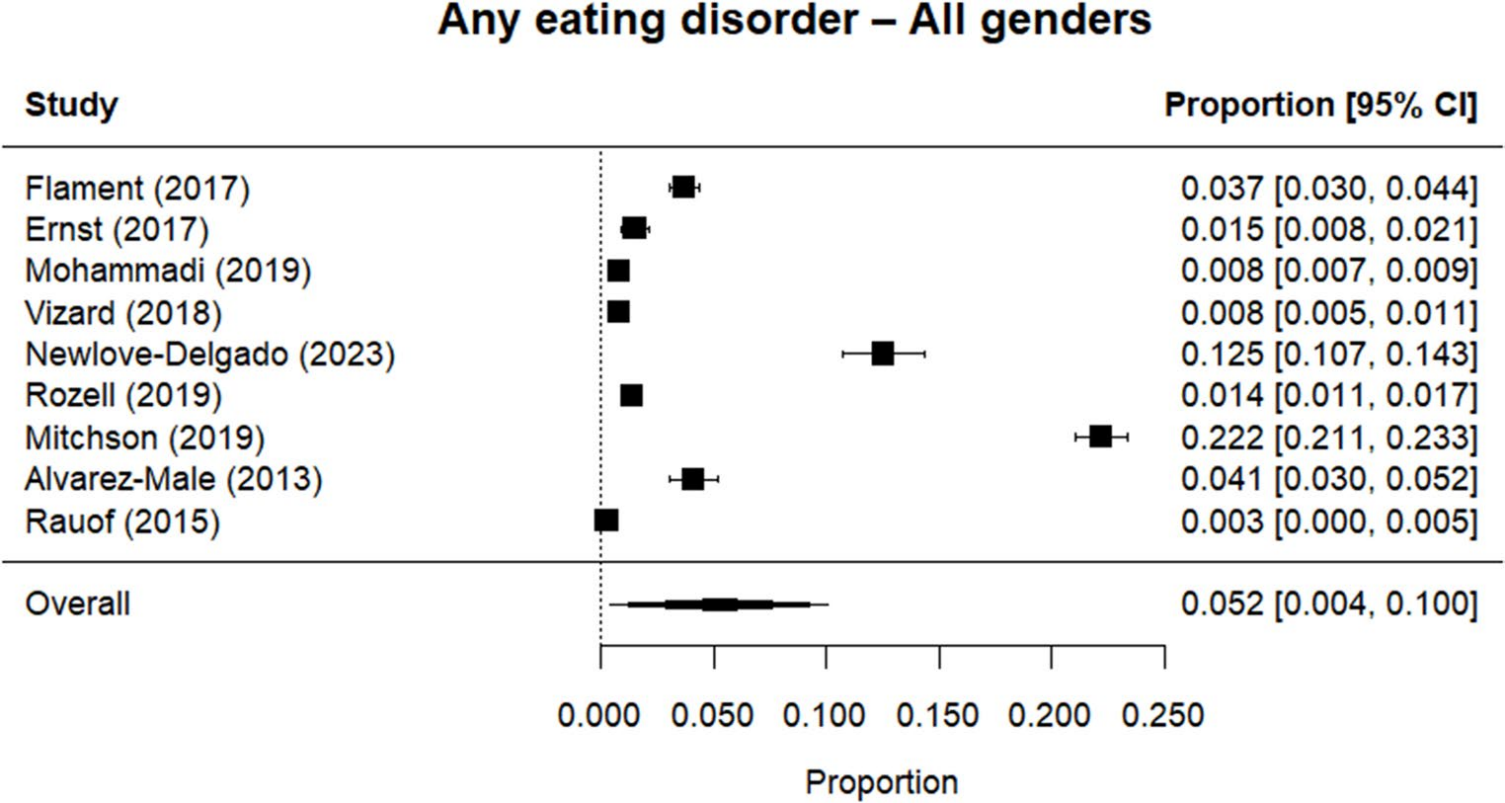
Forest plot of the point prevalence of any eating disorders – all genders

**Fig. 5:**
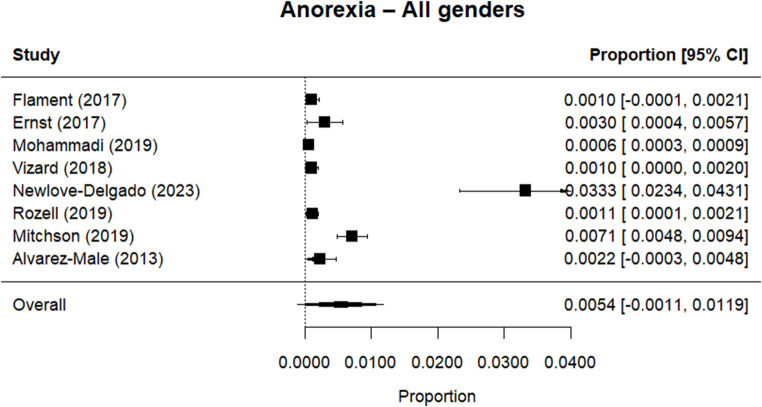
Forest plot of the point prevalence of anorexia – all genders

**Fig. 6:**
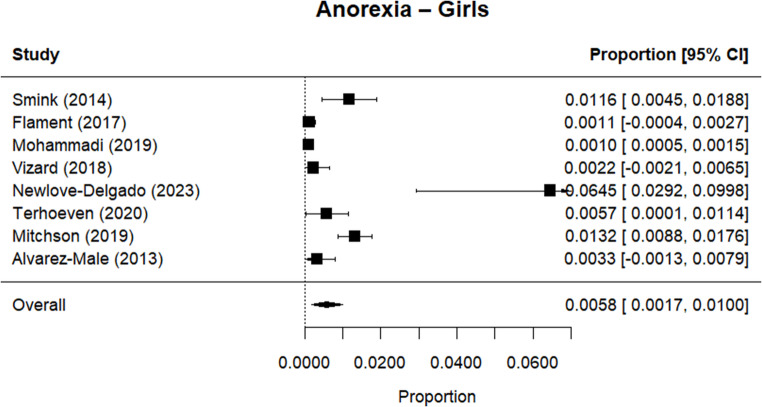
Forest plot of the point prevalence of anorexia – girls

**Fig. 7:**
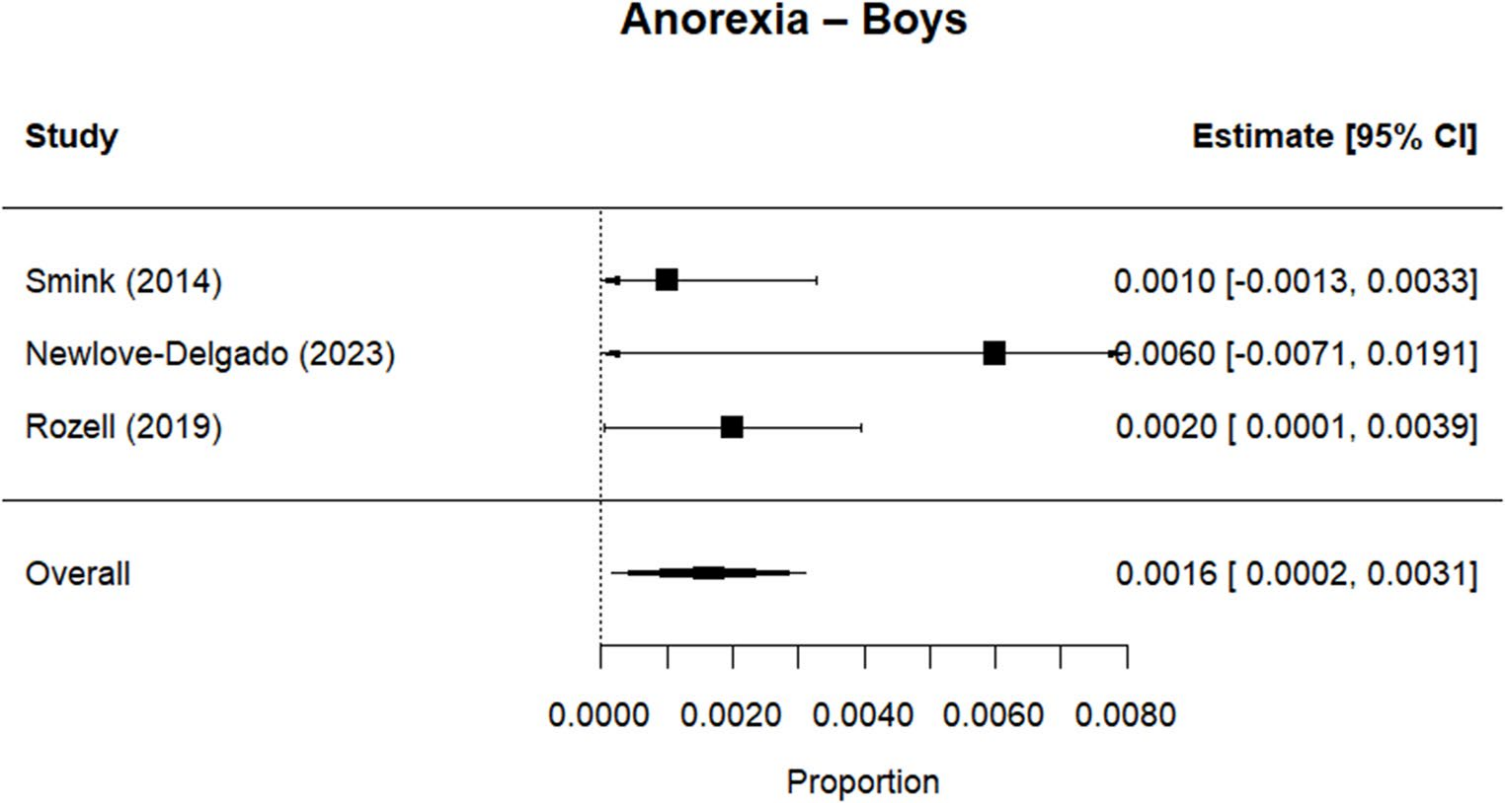
Forest plot of the point prevalence of anorexia – boys

**Fig. 8:**
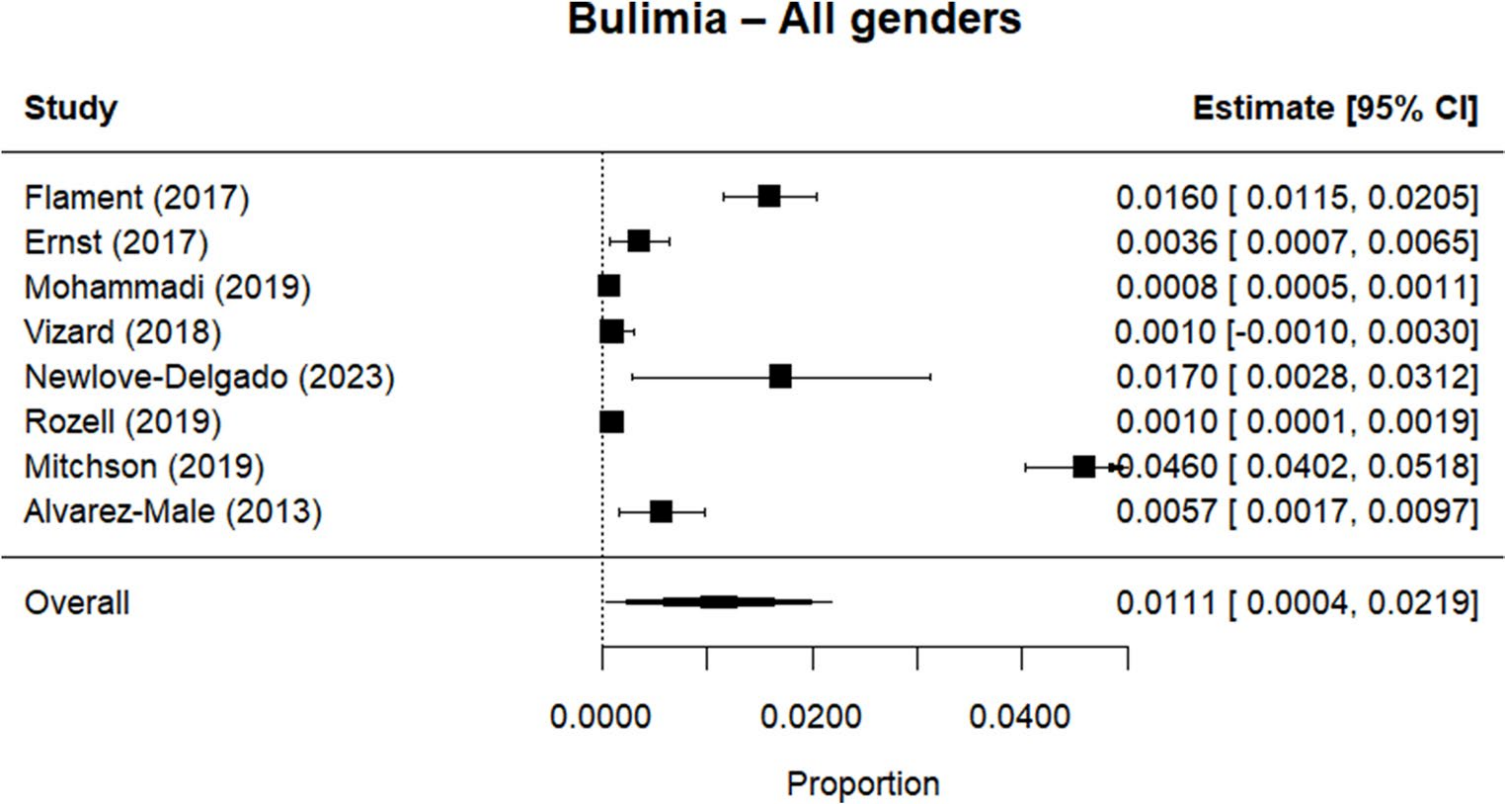
Forest plot of the point prevalence of bulimia – all genders

**Fig. 9:**
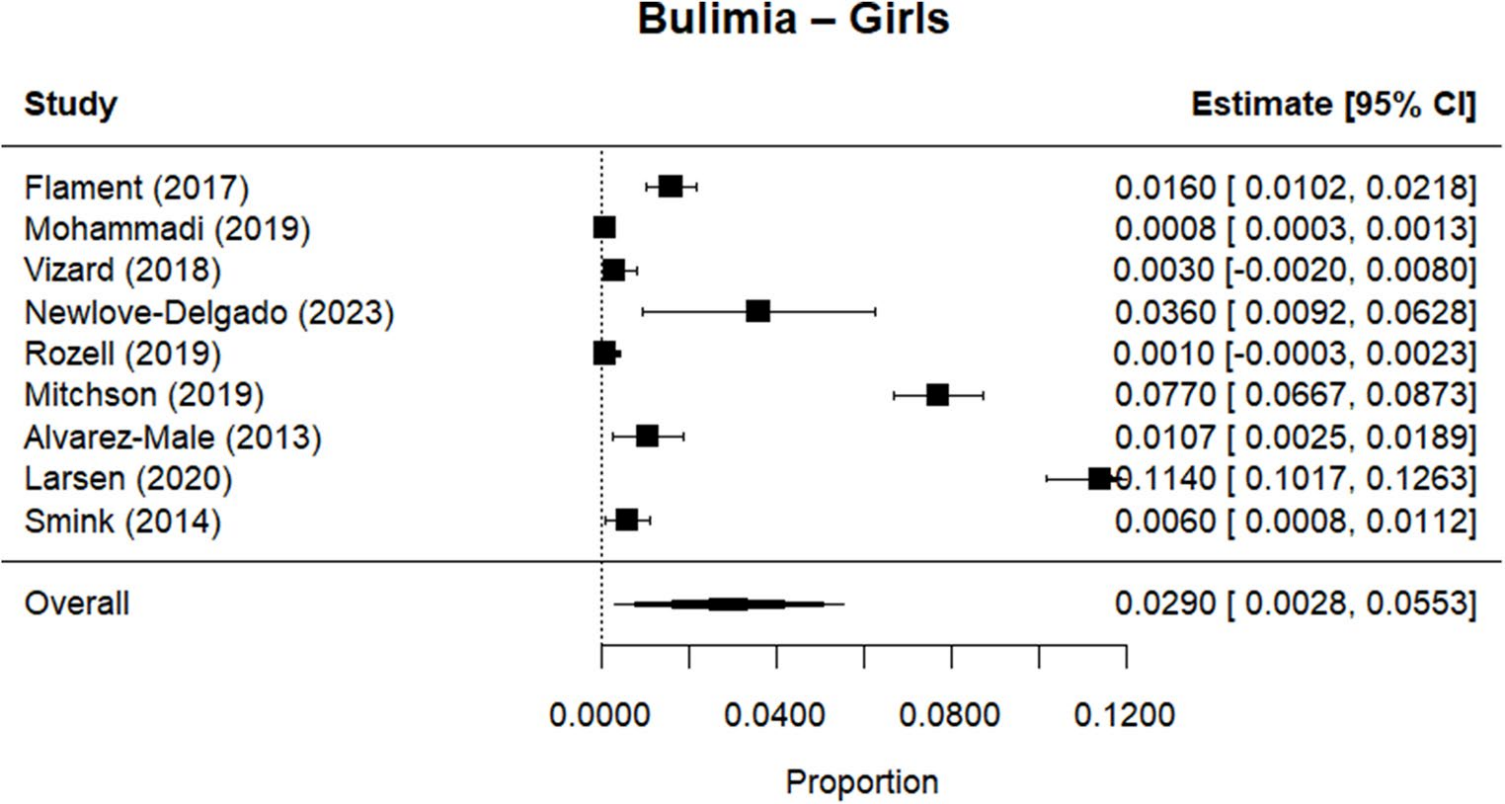
Forest plot of the point prevalence of bulimia – girls

**Fig. 10:**
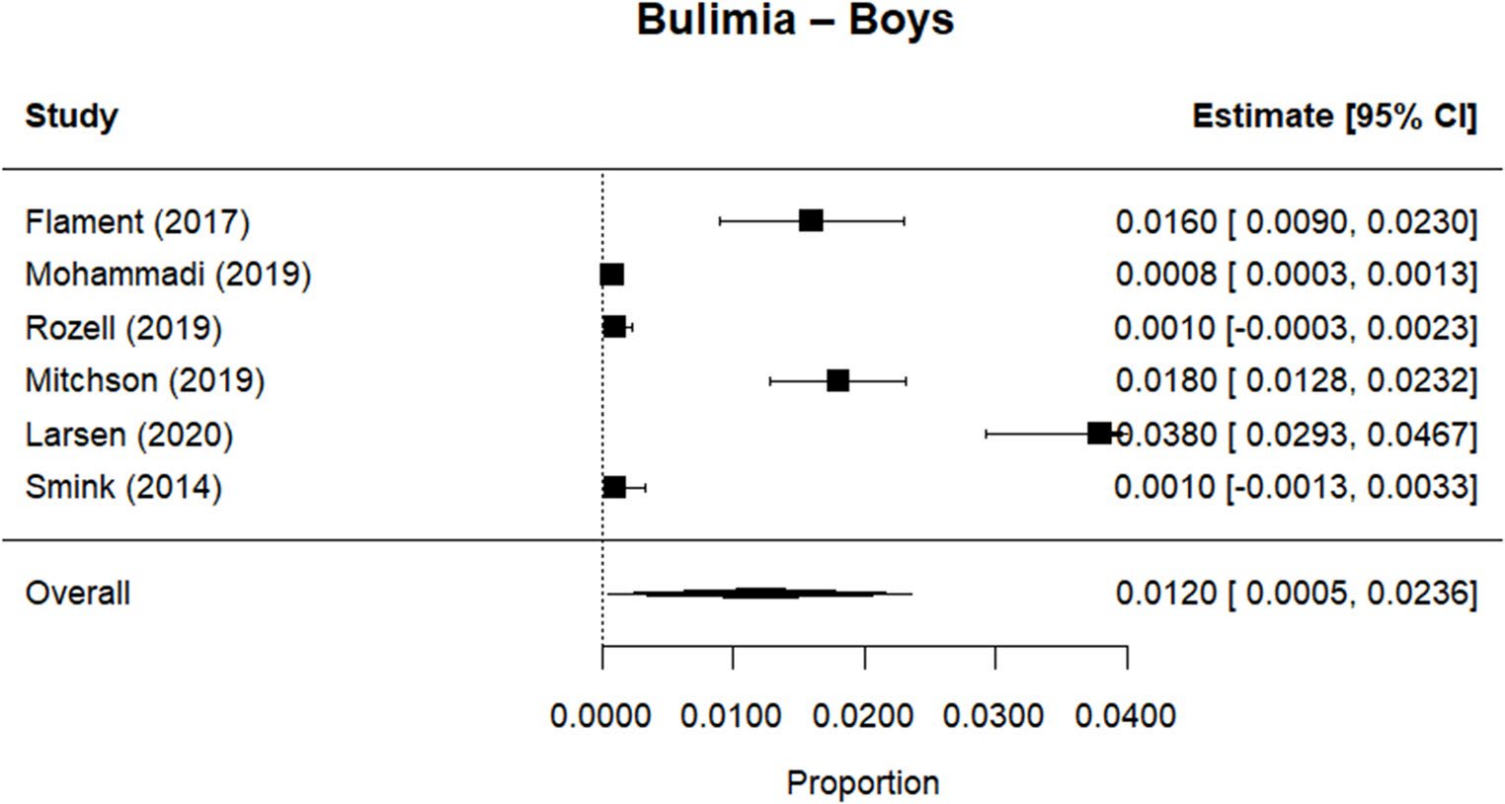
Forest plot of the point prevalence of bulimia – boys

**Fig. 11:**
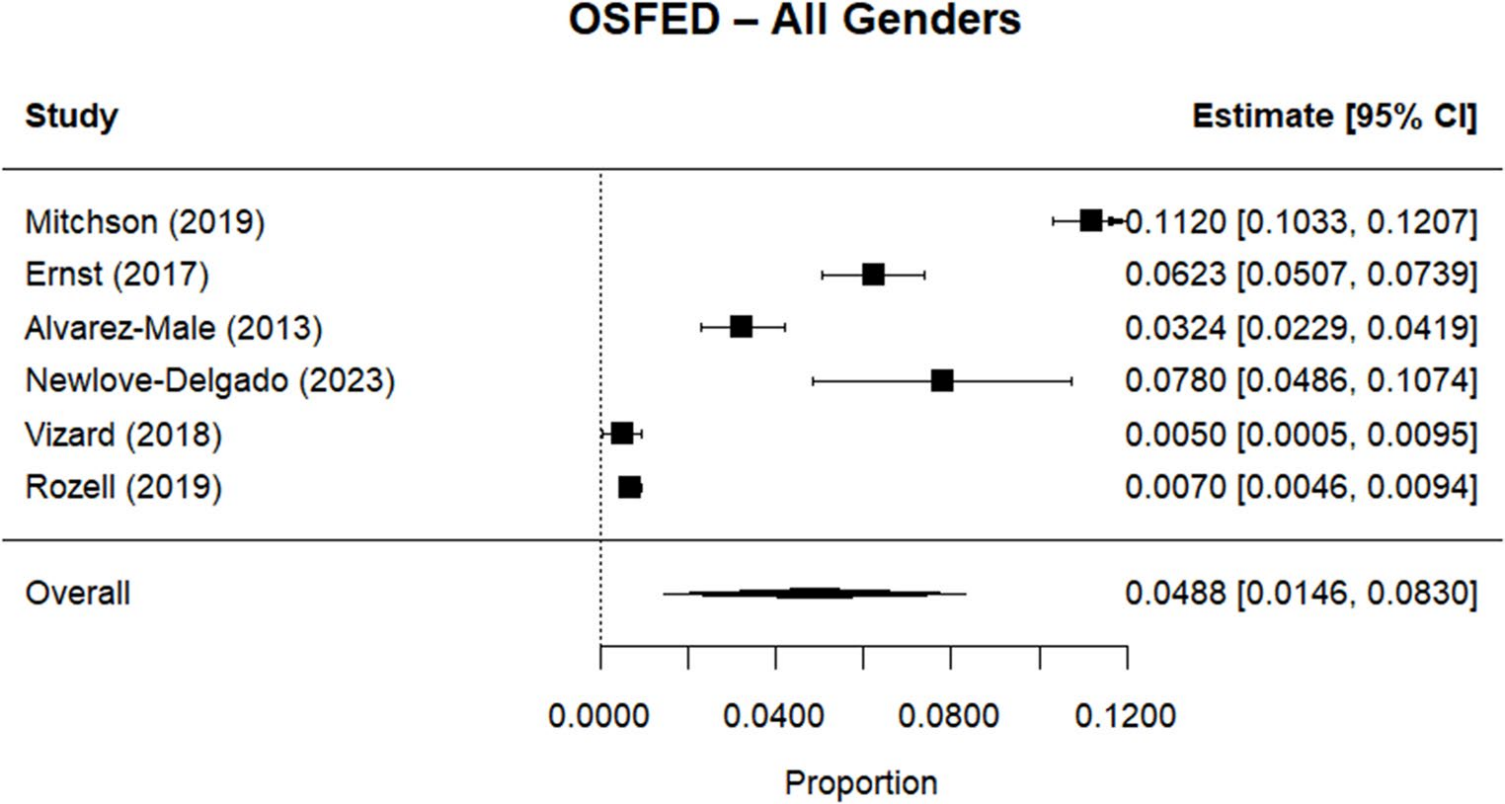
Forest plot of the point prevalence of OSFED – all genders

**Fig. 12:**
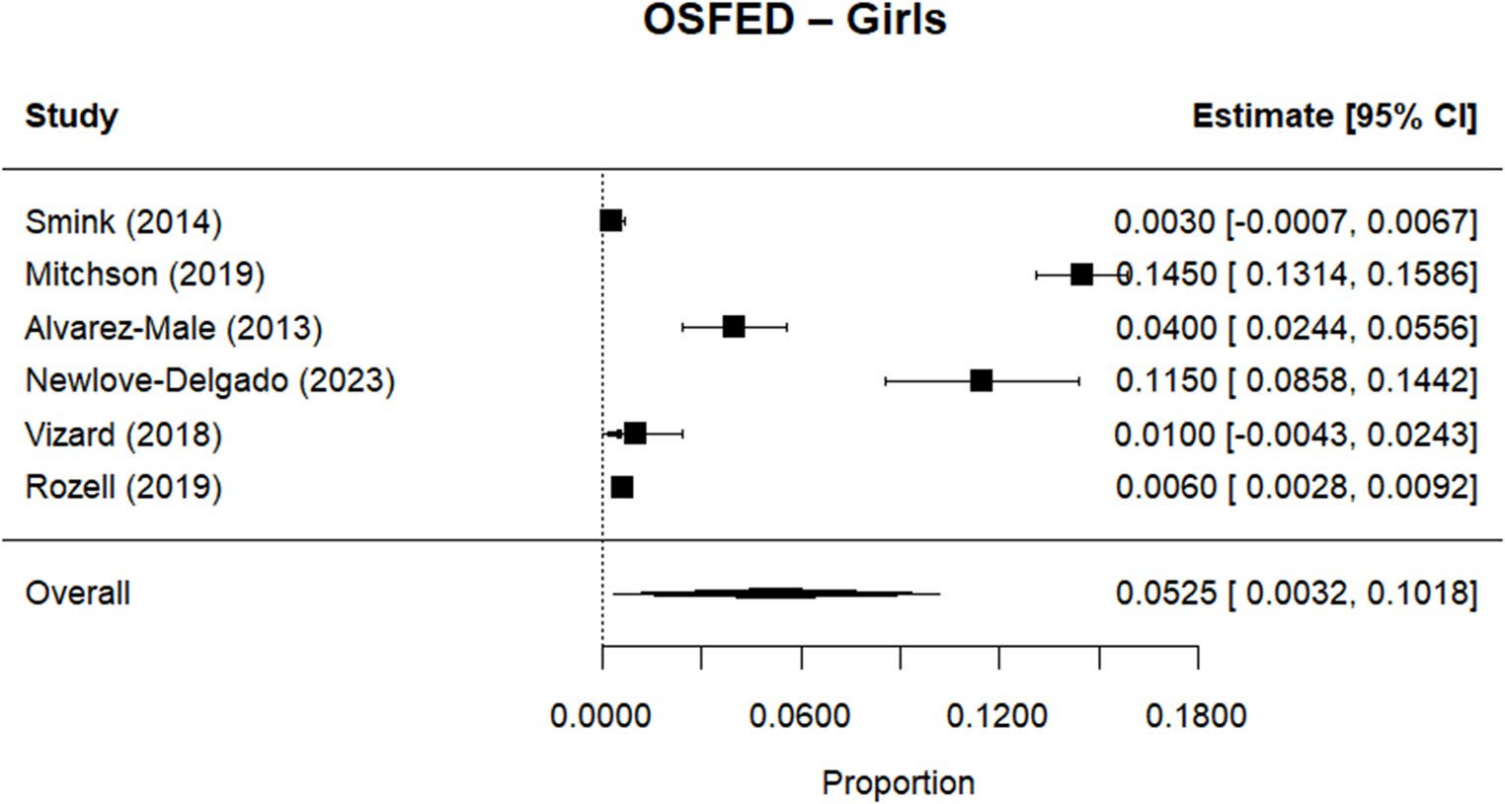
Forest plot of the point prevalence of OSFED – girls

**Fig. 13:**
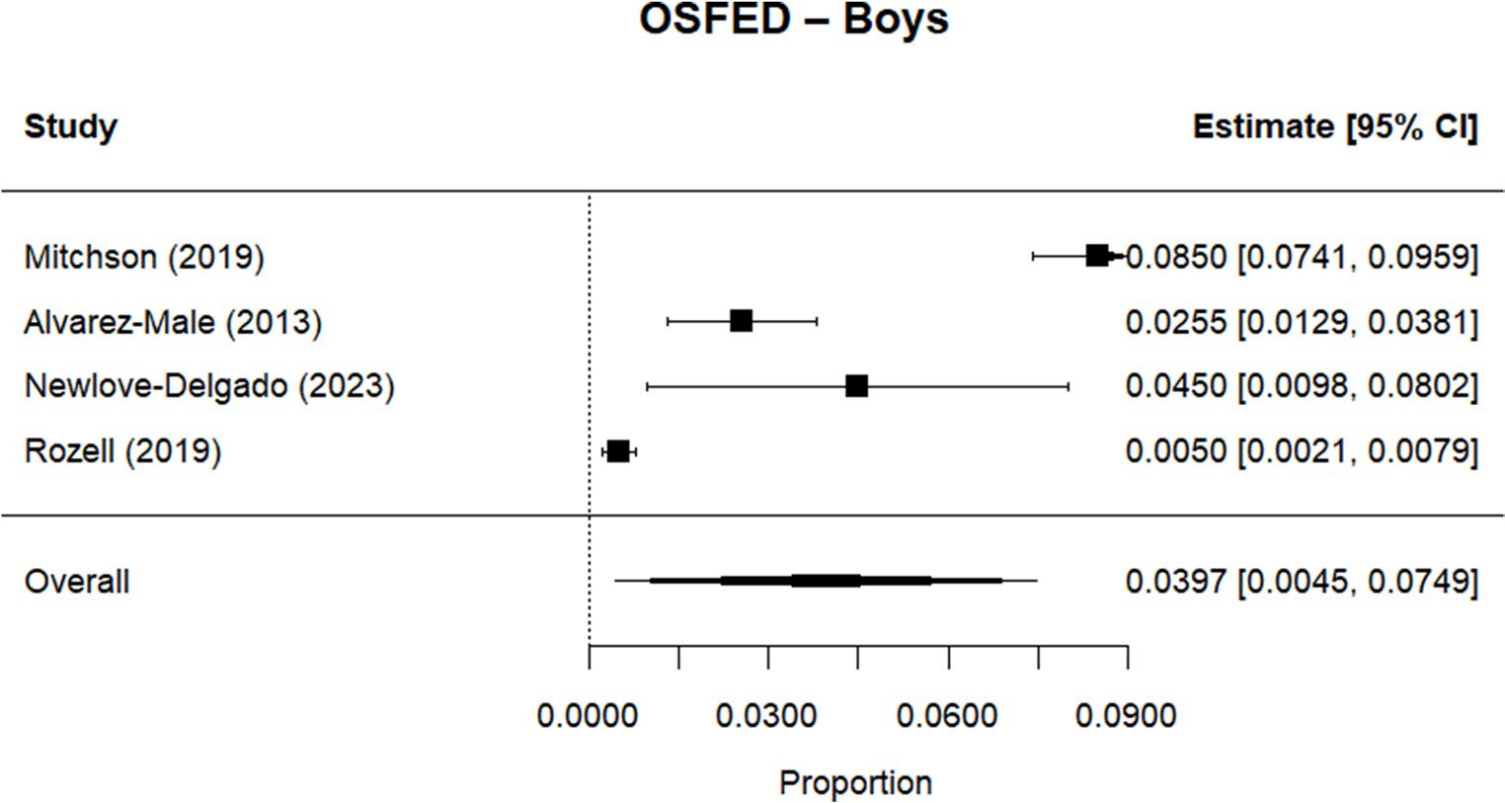
Forest plot of the point prevalence of OSFED – boys

 Random effects meta-analysis estimated a worldwide point-prevalence of any ED of 5.23% (95% CI 0.41–10.05; k = 9); pooled sample size = 50035 (see Figs. 3 and 4), but significant between study heterogeneity (I^2^> 99.95%, Q (df = 8) = 1635.16, p-value < 0.0001). Indeed, all meta-analyses conducted revealed high levels of heterogeneity (see Figs. 3, 4, 5, 6, 7, 8, 9, 10, 11, 12 and 13). The most common type of ED was OSFED 4.88% (95% CI 1.46–8.30; k = 6); pooled sample size = 13487; I^2^= 99.42%, Q (df = 5) = 634.1612, p-value < 0.0001(see Figs. 11, 12 and 13) and the least common was anorexia nervosa 0.54% (95% CI - 0.1–1.17; k = 8); pooled sample size = 48045; I^2^= 99.64%, Q (df = 7) = 74.8720, p-value < 0.0001 (see Figs. 5, 6 and 7). The prevalence of bulimia across all genders was 1.11% (95% CI 0.04–2.19; k = 8); pooled sample size = 43980; I^2^= 99.74%, Q (df = 7) = 291.4442, p-value < 0.0001 (see Figs. 8, 9 and 10).

 All EDs were more common in girls as shown in Fig. 3, but there was not a significant statistical difference in prevalence estimates in girls and boys, as confidence intervals overlapped. For any ED, the pooled prevalence among girls was 7.12% (95% CI 0.0–15.64; k = 7); pooled sample size = 23139; ​​I^2^= 99.94%, Q (df = 6) = 1280.0238, p-value < 0.0001 while for boys it was 2.99% (95% CI 0.0–6.21; k = 7); pooled sample size = 21947; ​​I^2^= 99.79%, Q (df = 6) = 378.5259, p-value < 0.0001. Moreover, the pooled prevalence of anorexia nervosa in girls was 0.60% (95% CI 0.19–1.01; k = 8); pooled sample size = 21011; ​​I^2^= 94.32%, Q (df = 7) = 53.4005, p-value < 0.0001 while for boys it was 0.16% (95% CI 0.02–0.31; k = 3); pooled sample size = 3199; I^2^= 0.00%, Q (df = 2) = 0.8575, p-value = 0.6513. For bulimia, the pooled prevalence among girls was 2.90% (95% CI 0.28–5.53; k= 9); pooled sample size = 25,077; ​​I^2^= 99.90%, Q (df = 8) = 574.9276, p-value < 0.0001) while for boys it was 1.20%, (95% CI 0.05–2.36; k = 6); pooled sample size = 21947; ​​I^2^= 99.58%, Q (df = 5) = 128.5795, p-value < 0.0001. Complete results for the meta-regression are shown in Table S3. Only geographic location (R^2^= 81.46%, p-value < 0.0001) and diagnostic interview (R^2^= 85.60%, p-value < 0.0001) were significant predictors of heterogeneity. Studies conducted in Oceania had significantly higher prevalence estimates for EDs compared with the other regions (North America, Europe and the Middle East). However, some regions were underrepresented (i.e. the Middle East compared with Europe), and results should be considered exploratory. We also performed a meta-regression to analyse the associations between year of data collection and prevalence. Year of data collection was not a significant factor (p-value = 0.2162).

## Discussion

 The current meta-analysis included data from over 50,000 CYP worldwide and estimated the point prevalence of any ED among CYP to be 5.23% (95% CI 0.41 to 10.05), which approximates to at least one in every classroom and indicates a significant level of need. We found that the most prevalent ED in children and young people in the community was OSFED (Other Specified Eating Disorder), followed by Bulimia Nervosa and Anorexia Nervosa.A previous meta-analysis also reported OSFED as the most common eating disorder, however it included studies of both adults and adolescents and did not report heterogeneity estimates [36].

 We only included studies which used ED diagnostic criteria that stress clinical impairment with the obvious implication for clinical services, unlike previous meta-analyses which have estimated subthreshold ED symptoms or disordered eating [12, 37].Notably, the largest study measuring the impact of eating disorders - the global burden of disease study - only included estimates for anorexia and bulimia both in the GBD 2019 and in the GBD 2021, yet in adults there is substantial evidence pointing out OSFED is more prevalent, which is also consistent with our findings in children and adolescents [38]. In 2021, an analysis estimated that the GBD 2019 underestimated the prevalence of eating disorders by 41·9 million cases (95% uncertainty interval 27·9–59·0) by not including OSFED and binge eating disorders [38]. These alarming numbers suggest the burden and prevalence of EDs worldwide is significantly underestimated by the current available evidence.

 We could not analyse whether the prevalence of eating disorders increased post COVID-19, because only one included study collected data after 2020. All the other studies were conducted before the COVID-pandemic, which highlights the importance of including rigorous measurement of eating disorders in children and young people in national surveys. Between the GBD 2019 and the GBD 2021, the prevalence estimates for the 10–24-year-old age group did not change substantially; 0.38 (CI 0.26–054) to 0.37 (CI 0.26–054) respectively [39, 40]. Considering that the most common eating disorder in adults and the current study was not included in those estimates, it is difficult to ascertain if the prevalence of eating disorders substantially changed post covid. In our own findings, due to the small number of studies measuring ED prevalence in young people in community settings, the confidence intervals for the pooled estimates in the meta-analysis are wide and so a true increase over time may be obscured. In the meta-regression, we could only include nine studies, therefore we believe it is not categorically possible to affirm ED prevalence in youth did not vary over time since 2013. Further, two thirds of the included studies had a low or moderate risk of bias, leaving one third with seriously flawed methodology.

 In the meta-regression, only geographical location and diagnostic interview were significant for explaining differences in prevalence. EDs, and particularly body self-perceptions, are heavily influenced by culture and more studies across different countries are required to explore this phenomenon further. For example, there were no high-quality studies from certain parts of the world like South America or Africa.

 The present work has two major implications. More population prevalence studies with robust methodology are urgently needed so we can inform service planning and monitor change in eating disorder prevalence over time. It is particularly important to include validated measurements of OSFED in national surveys and large-scale studies, such as the global burden of disease study. Our findings suggest that we are currently underestimating the burden of eating disorders in children and young people.

 Our results can also inform the design of those future studies. Like others, we found that the diagnostic interview selected significantly influenced prevalence estimates, while study design, sampling frame and informant did not [41, 42]. Future surveys should be more consistent in their methodology so the results can be directly comparable, while larger samples would deliver more precise estimates. Notably, only two studies [30, 31] included more than 10,000 participants while four (27%) included fewer than two thousand. Besides weighting surveys to account for non-response and survey design, it is also important to engage or oversample traditionally excluded groups to improve representation and generalisability. Surveys should gather data systematically on trans and gender diverse young people, who are at increased risk to present disordered eating, [43, 44] but who were explicitly included in only one studyin the current review [30].

The literature on eating disorders has focused mostly on prevalence in clinical settings [36]. Nonetheless, accurate community prevalence estimates in CYP are much needed for public health monitoring, service commissioning and disease-burden estimates, as even the best resourced services are unlikely to identify and treat all CYP with eating disorders in the community. Arguably, given the rapid increase in clinical presentations during the Covid-19 pandemic, knowledge of population prevalence is particularly important currently. For instance, a recent systematic review and meta-analysis [16] reported an overall increase worldwide of healthcare utilisation by CYP with eating disorders, however, in public health systems such as the National Health Service (NHS), without accurate and high-quality data is difficult for policy makers to adequately resource services and manage waiting list times. Moving forward, is important to raise awareness among practitioners working with CYP who might be particularly at risk as it is unlikely that at the initial stages of an ED, they will come forward on their own [6]. The Royal College of Psychiatrists surveyed all medical schools and postgraduate training programs in the UK and reported that most doctors in the UK were never assessed on their knowledge of eating disorders during their entire training, while only a few medical students and trainees had the opportunity to improve their clinical skills on eating disorders. The average teaching time around the condition for UK medical students is 2 hours [45]. We need to ensure that health professionals have access to training and advice to ensure timely referral for suspected eating disorder cases.

 The present work also has several limitations. First, apart from the pooled prevalence of anorexia among boys, all results were heterogeneous and hence, should be interpreted with caution. We performed a meta-regression, but significant unexplained heterogeneity remained.

High levels of heterogeneity might be explained by the diverse nature of different types of EDs, which other than dysfunctional eating behaviour have varied clinical features. However, this would not explain the high heterogeneity found within disorder-specific estimates. Meta-analysis of pooled prevalence estimates in mental health sciences have traditionally reported high heterogeneity estimates [41, 42].

Moreover, we lacked time and resources to double screen 100% of the 17346 titles and abstracts that we retrieved. As our primary screener who viewed 100% of records has considerable experience, we supplemented database searches with hand searches, and we achieved excellent chance-corrected agreement across all screeners – evidenced by the Cohen’s Kappa for title and abstract screening of 0,85. Hence, we are confident that we have not missed key references [46].

## Conclusions

In conclusion, EDs are common among CYP in the community, with OSFED being the most common type but are understudied particularly at population level. To the best of our knowledge, this is the first systematic review, meta-analysis, and meta-regression on these disorders among this age group. Significant heterogeneity points to the importance of the field settling on the best diagnostic assessment and improved methodological rigour for future population surveys. Our findings have implications for mental health policy and ED awareness worldwide, particularly for the systematic inclusion and measurement of OSFED in future studies.

## Supplementary Information

Below is the link to the electronic supplementary material.

Supplementary Material 1 (DOCX 43.2 KB)

## Data Availability

Data for the searches and protocol is provided within the manuscript, supplementary information files and in the registered PROSPERO protocol. Data extraction tables are available upon request. All code used for the analysis is deposited on git.hub.

## Abbreviations

ED: Eating disorder
CYP: Children and young people

## References

[1] 1. Saulle, R., De Sario, M., Bena, A., Capra, P., Culasso, M., Davoli, M., et al. (2022). School closures and mental health, wellbeing, and health behaviors among children and adolescents during the second COVID-19 wave: A systematic review of the literature. Epidemiologia e Prevenzione, 46(5–6), 333–352. 10.19191/EP22.5-6.A542.089 36384255

[2] 2. Newlove-Delgado, T., Marcheselli, F., Williams, T., Mandalia, D., Dennes, M., McManus, S., et al. (2023). Mental health of children and young people in England, 2023. NHS England, Leeds.

[3] Taquet M, Geddes JR, Luciano S, Harrison PJ (2022) Incidence and outcomes of eating disorders during the COVID-19 pandemic. The Lancet Child & Adolescent Health 6(3):231–232

[4] Solmi F, Downs JL, Nicholls D (2021) Eating disorders in young people: trends, challenges, and opportunities. The Lancet Child & Adolescent Health 5(2):88–8933340468

[5] Ward JL et al (2025) Admission to acute medical wards for mental health concerns among children and young people in England from 2012 to 2022: a cohort study. The Lancet Child & Adolescent Health 9(2):112–120 39855751 10.1016/S2352-4642(24)00333-X

[6] Treasure J, Duarte TA, Schmidt U (2020) Eating disorders. Lancet 395(10227):899–911 32171414 10.1016/S0140-6736(20)30059-3

[7] American Psychiatric Association (2013) Diagnostic and statistical manual of mental disorders: DSM-5, 5th ed. American Psychiatric Publishing, Arlington, VA

[8] 8. ICD-11. (n.d.). Retrieved from https://icd.who.int/en

[9] Stice E, Marti CN, Rohde P (2013) Prevalence, incidence, impairment, and course of the proposed DSM-5 eating disorder diagnoses in an 8-year prospective community study of young women. J Abnorm Psychol 122(2):445–457 23148784 10.1037/a0030679 PMC3980846

[10] Franko DL, Becker AE, Thomas JJ, Herzog DB (2007) Cross-ethnic differences in eating disorder symptoms and related distress. Int J Eat Disord 40(2):156–164 17080449 10.1002/eat.20341

[11] Nicholls D (2023) Editorial perspective: a perfect storm—how and why eating disorders in young people have thrived in lockdown and what is happening to address it. J Child Psychol Psychiatry 64(2):335–338 35902107 10.1111/jcpp.13676 PMC10087223

[12] López-Gil JF, García-Hermoso A, Smith L, Firth J, Trott M, Mesas AE et al (2023) Global proportion of disordered eating in children and adolescents: a systematic review and meta-analysis. JAMA Pediatr 177(4):363–372 36806880 10.1001/jamapediatrics.2022.5848 PMC9941974

[13] Culbert KM, Racine SE, Klump KL (2015) Research review: what we have learned about the causes of eating disorders—a synthesis of sociocultural, psychological, and biological research. J Child Psychol Psychiatry 56(11):1141–1164 26095891 10.1111/jcpp.12441

[14] Jacobi C, Hayward C, de Zwaan M, Kraemer HC, Agras WS (2004) Coming to terms with risk factors for eating disorders: application of risk terminology and suggestions for a general taxonomy. Psychol Bull 130(1):19–65 14717649 10.1037/0033-2909.130.1.19

[15] 15. Yamamiya, Y., & Stice, E. (2023). Risk factors that predict future onset of anorexia nervosa, bulimia nervosa, binge eating disorder, and purging disorder in adolescent girls. Behavior Therapy. 10.1016/j.beth.2023.10.002 PMC11211638 38937045

[16] 16. Madigan, S., Vaillancourt, T., Dimitropoulos, G., Premji, S., Kahlert, S. M., Zumwalt, K., Korczak, D. J., von Ranson, K. M., Pador, P., Ganshorn, H., & Neville, R. D. (2024). A Systematic Review and Meta-Analysis: Child and Adolescent Healthcare Utilization for Eating Disorders During the COVID-19 Pandemic. Journal of the American Academy of Child and Adolescent Psychiatry. 10.1016/j.jaac.2024.02.009 38431196

[17] Solmi F, Downs JL (2021) A global perspective on eating disorders. Curr Opin Psychiatry 34(6):528–534

[18] 18. Page, M. J., McKenzie, J. E., Bossuyt, P. M., Boutron, I., Hoffmann, T. C., Mulrow, C. D., et al. (2021). The PRISMA 2020 statement: An updated guideline for reporting systematic reviews. *BMJ, 2021*, n71. 10.1136/bmj.n71 PMC8005924 33782057

[19] Hoy D, Brooks P, Woolf A, Blyth F, March L, Bain C et al (2012) Assessing risk of bias in prevalence studies: modification of an existing tool and evidence of interrater agreement. J Clin Epidemiol 65(9):934–939 22742910 10.1016/j.jclinepi.2011.11.014

[20] Glazer KB, Sonneville KR, Micali N, Swanson SA, Crosby R, Horton NJ et al (2019) The course of eating disorders involving bingeing and purging among adolescent girls: prevalence, stability, and transitions. J Adolesc Health 64(2):165–171 30509766 10.1016/j.jadohealth.2018.09.023 PMC10535941

[21] Smink FRE, van Hoeken D, Oldehinkel AJ, Hoek HW (2014) Prevalence and severity of DSM-5 eating disorders in a community cohort of adolescents. Int J Eat Disord 47(6):610–619 24903034 10.1002/eat.22316

[22] Terhoeven V, Lemhöfer C, Rief W (2020) Diagnostic accuracy of self-report questionnaires for eating disorders in primary care: a systematic review. J Psychosom Res 135:110173

[23] Heydari Yazdi AS, Eslamzadeh M, Mohammadi MR, Khaleghi A, Hooshyari Z, Moharreri F et al (2020) A survey of psychiatric disorders and their comorbidities in children and adolescents. Galen Medical Journal 9:e1714 34466575 10.31661/gmj.v9i0.1714 PMC8344126

[24] Hippel VTP (2015) The heterogeneity statistic I² can be biased in small meta-analyses. BMC Med Res Methodol 15(1):1–8 25880989 10.1186/s12874-015-0024-z PMC4410499

[25] Álvarez-Malé ML, Bautista Castaño I, Serra Majem L (2015) [Prevalence of eating disorders in adolescents from Gran Canaria]. Nutr Hosp 31(5):2283–2288 25929405 10.3305/nh.2015.31.5.8583

[26] Flament MF, Buchholz A, Henderson K, Obeid N, Maras D, Schubert N et al (2015) Comparative distribution and validity of DSM-IV and DSM-5 diagnoses of eating disorders in adolescents from the community. Eur Eat Disord Rev 23(2):100–110 25524758 10.1002/erv.2339

[27] Ernst V, Bürger A, Hammerle F (2017) Prevalence and severity of eating disorders: a comparison of DSM-IV and DSM-5 among German adolescents. Int J Eat Disord 50(11):1255–1263 28963857 10.1002/eat.22780

[28] 28. Vizard, T., Sadler, K., Ford, T., et al. (2018). *Mental health of children and young people in England, 2017: Survey design and methods report*.

[29] Larsen JT, Munk-Olsen T, Bulik CM, Thornton LM, Koch SV, Mortensen PB et al (2017) Early childhood adversities and risk of eating disorders in women: a Danish register-based cohort study. Int J Eat Disord 50(12):1404–1412 29105808 10.1002/eat.22798

[30] Mitchison D, Mond J, Bussey K, Griffiths S, Trompeter N, Lonergan A et al (2020) DSM-5 full syndrome, other specified, and unspecified eating disorders in Australian adolescents: prevalence and clinical significance. Psychol Med 50(6):981–990 31043181 10.1017/S0033291719000898

[31] Mohammadi MR, Mostafavi SA, Hooshyari Z, Khaleghi A, Ahmadi N, Molavi P et al (2020) Prevalence, correlates, and comorbidities of feeding and eating disorders in a nationally representative sample of Iranian children and adolescents. Int J Eat Disord 53(3):349–361 31742760 10.1002/eat.23197

[32] Rauof M, Ebrahimi H, Asghari Jafarabadi M, Malek A, Babapour Kheiroddin J (2015) Prevalence of eating disorders among adolescents in the northwest of Iran. Iran Red Crescent Med J 17(10):e19331 26568851 10.5812/ircmj.19331 PMC4640058

[33] Rozzell K, Moon DY, Klimek P, Brown T, Blashill AJ (2019) Prevalence of eating disorders among US children aged 9 to 10 years. JAMA Pediatr 173(1):100–101 30476983 10.1001/jamapediatrics.2018.3678 PMC6583451

[34] 34. Silén, Y., Sipilä, P. N., Raevuori, A., Mustelin, L., Marttunen, M., Kaprio, J., et al. (2020). DSM-5 eating disorders among adolescents and young adults in Finland: A public health concern. *International Journal of Eating Disorders, 53*(5), 520–531. 10.1002/eat.23236 31999001

[35] Chen, Y. L., Chen, W. J., Lin, K. C., Shen, L. J., & Gau, S. S. (2019). Prevalence of DSM-5 mental disorders in a nationally representative sample of children in Taiwan: methodology and main findings. *Epidemiology and psychiatric sciences*, *29*, e15. 10.1017/S2045796018000793 10.1017/S2045796018000793 PMC8061245 30696515

[36] Galmiche, M., Déchelotte, P., Lambert, G., & Tavolacci, M. P. (2019). Prevalence of eating disorders over the 2000–2018 period: A systematic literature review. *American Journal of Clinical Nutrition, 109*(5), 1402–1413 10.1093/ajcn/nqy342 31051507

[37] Paranjothy, S. M., & Wade, T. D. (2024). A meta-analysis of disordered eating and its association with self-criticism and self-compassion. *International Journal of Eating Disorders*. 10.1002/eat.24166 38366726

[38] Santomauro, D. F., Melen, S., Mitchison, D., Vos, T., Whiteford, H., & Ferrari, A. J. (2021). The hidden burden of eating disorders: an extension of estimates from the Global Burden of Disease Study 2019. *The lancet. Psychiatry*, *8*(4), 320–328. 10.1016/S2215-0366(21)00040-7 10.1016/S2215-0366(21)00040-7 PMC7973414 33675688

[39] GBD 2019 Fracture Collaborators (2021). Global, regional, and national burden of bone fractures in 204 countries and territories, 1990–2019: a systematic analysis from the Global Burden of Disease Study 2019. *The lancet. Healthy longevity*, *2*(9), e580–e592. 10.1016/S2666-7568(21)00172-0 10.1016/S2666-7568(21)00172-0 PMC8547262 34723233

[40] GBD 2021 Causes of Death Collaborators (2024). Global burden of 288 causes of death and life expectancy decomposition in 204 countries and territories and 811 subnational locations, 1990–2021: a systematic analysis for the Global Burden of Disease Study 2021. *Lancet (London, England)*, *403*(10440), 2100–2132. 10.1016/S0140-6736(24)00367-2 10.1016/S0140-6736(24)00367-2 PMC11126520 38582094

[41] Guilherme Polanczyk, M. D., Maurício Silva de Lima, M. D., Bernardo Lessa Horta, M. D., Joseph Biederman, M. D., & Luis Augusto Rohde, M. D. (2007). The worldwide prevalence of ADHD: A systematic review and metaregression analysis. *American Journal of Psychiatry*. Retrieved from https://ajp.psychiatryonline.org/doi/10.1176/ajp.2007.164.6.942 10.1176/ajp.2007.164.6.942 17541055

[42] Polanczyk, G. V., Salum, G. A., Sugaya, L. S., Caye, A., & Rohde, L. A. (2015). Annual research review: A meta-analysis of the worldwide prevalence of mental disorders in children and adolescents. *Journal of Child Psychology and Psychiatry, 56(3)*, 345–365. 10.1111/jcpp.12381 10.1111/jcpp.12381 25649325

[43] Heiden-Rootes, K., Linsenmeyer, W., Levine, S., Oliveras, M., & Joseph, M. (2023). A scoping review of research literature on eating and body image for transgender and nonbinary youth. *Journal of Eating Disorders, 11*(1), 168. 10.1186/s40337-023-00853-5 PMC10517525 37740228

[44] Rasmussen, S. M., Dalgaard, M. K., Roloff, M., Pinholt, M., Skrubbeltrang, C., Clausen, L., et al. (2023). Eating disorder symptomatology among transgender individuals: A systematic review and meta-analysis. *Journal of Eating Disorders, 11*(1), 84. 10.1186/s40337-023-00806-y PMC10214585 37237320

[45] Ayton, A., & Ibrahim, A. (2018). Does UK medical education provide doctors with sufficient skills and knowledge to manage patients with eating disorders safely? *Postgraduate Medical Journal, 94*(1113), 374–380. 10.1136/postgradmedj-2018-135658 10.1136/postgradmedj-2018-135658 29866707

[46] Waffenschmidt, S., Knelangen, M., Sieben, W. et al. Single screening versus conventional double screening for study selection in systematic reviews: a methodological systematic review. *BMC Med Res Methodol* ***19***, *132 (2019).* 10.1186/s12874-019-0782-0 PMC6599339 31253092

