# Supplementary material for: The global prevalence of eating disorders in children and young people: a systematic review and meta-analysis
Source: Eur Child Adolesc Psychiatry. 2026 Jan 27;35(4):1093–106. doi: 10.1007/s00787-025-02933-0 (PMC13219161; doi:10.1007/s00787-025-02933-0)
Supplement: Supplementary file 1 — Supplementary Material 1 (DOCX 43.2 KB) [file 787_2025_2933_MOESM1_ESM.docx]

**Supporting Information**

**The global prevalence of eating disorders in children and young people: a systematic review and meta-analysis**

Clara Faria MD^1^, Kian Daneshi^2^, Aslihan Baser MSc^3^, Henrike Mauersberger ^4^, Abigail G-Medhin^5^, Emma Soneson PhD^6^, Simon White PhD^7*^, Joanna Anderson PhD^8*^ and Tamsin Ford FRCPsych PhD^9*^

^1^ Department of Psychiatry, University of Cambridge, ORCID: 0000-0002-1290-2349

^2^ School of Medicine, University of Sheffield, ORCID: 0009-0000-6478-7891

^3^ Department of Psychiatry, University of Cambridge, ORCID: 0009-0009-8468-3710

^4^ Department of Psychiatry, University of Cambridge, ORCID: 0009-0008-1261-7864

^5^ GKT School of Medical Education, Kings College London, UK, ORCID:  0000-0001-6928-8493

^6^ Department of Psychiatry, University of Oxford, ORCID: 0000-0003-1666-3012

^7^ Department of Psychiatry, University of Cambridge, ORCID: 0000-0001-8642-7037

^8^ Department of Psychiatry, University of Cambridge, ORCID: 0000-0002-0565-3735

^9^ Department of Psychiatry, University of Cambridge, ORCID: 0000-0001-5295-4904

^*^ Contributed equally as senior authors

**Correspondence to:**

Clara Gitahy Falcao Faria

Hershel Smith Building,

Forvie Site
Robinson Way
Cambridge Biomedical Campus
Cambridge CB2 0SZ

+44 07472 912238

Cgff2@cam.ac.uk

**Table S1.1 - Full Search Terms for Ovid/Medline**

|  | text words | controlled vocabulary (MeSH) |
| --- | --- | --- |
| Concept:  adolescents and children | adolescent* [tw] OR  teen* [tw] OR  child* [tw] OR  “Child, preschool” [tw] OR  youth [tw] OR  “Young person” [tw] OR  “Young people” [tw] | Adolescent [mh] OR  Child [mh] OR  “Child, preschool” [mh] |
| Concept:  prevalence studies | “Prevalence study” [tw] OR  “Prevalence studies” [tw] OR  prevalence [tw] OR  “cross-sectional studies” [tw] OR  incidence [tw] OR  epidemiolog* [tw] OR  “Probability Sample” [tw] | prevalence [mh]  OR  “cross-sectional studies” [mh]  OR  epidemiolog* [mh] |
| Concept:  Eating disorders | “Feeding and eating disorders” [tw] OR  “Feeding and Eating Disorders of Childhood” [tw] OR  “Atypical anorexia” [tw] OR  Anorexia [tw] OR  “Anorexia nervosa” [tw] OR  ARFID [tw] OR  Bulimia [tw] OR  “Bulimia nervosa” [tw] OR  “atypical bulimia” [tw] OR  ED [tw] OR  “Binge-eating disorder” [tw] OR  Bingeing [tw] OR  “Binge-eating” [tw] OR  “eating disorder” [tw] OR  “disordered eating” [tw] OR  neophobia [tw] | “Feeding and eating disorders” [mh]  OR “Feeding and Eating Disorders of Childhood” [mh] |

1. adolescent* [tw] OR teen* [tw] OR child* [tw] OR “Child, preschool” [tw] OR youth [tw] OR “young person” [tw] OR “young people” [tw]
2. Adolescent [mh] OR Child [mh] OR “Child, preschool” [mh]
3. “Prevalence study” [tw] OR “prevalence studies” [tw] OR prevalence [tw] OR “cross-sectional studies” [tw] OR incidence [tw] OR epidemiolog* [tw] OR “Probability Sample” [tw]
4. prevalence [mh] OR “cross-sectional studies” [mh] OR epidemiolog* [mh]
5. “Feeding and eating disorders” [tw] OR “Feeding and Eating Disorders of Childhood” [tw] OR “Atypical anorexia” [tw] OR Anorexia [tw] OR “Anorexia nervosa” [tw] OR ARFID [tw] OR Bulimia [tw] OR “Bulimia nervosa” [tw] OR “atypical bulimia” [tw] OR ED [tw] OR “Binge-eating disorder” [tw] OR Bingeing [tw] OR “Binge-eating” [tw] OR “eating disorder” [tw] OR “disordered eating” [tw] OR neophobia [tw]
6. “Feeding and eating disorders” [mh] OR “Feeding and Eating Disorders of Childhood” [mh]
7. #1 OR #2
8. #3 OR #4
9. #5 OR #6
10. #7 AND #8 AND #9

**S1.2 - Full Search Terms for EMBASE**

('child psychiatry' OR 'adolescence' OR 'child' OR 'juvenile' OR 'young people' OR 'adolescent'/de OR 'child'/de) AND ('prevalence' OR 'prevalence'/de OR 'cross-sectional study'/de OR 'incidence' OR 'cross-sectional study'/de OR 'probability sample' OR 'epidemiology' OR 'epidemiology'/de) AND ((feeding AND 'feeding behavior') OR 'eating disorder' OR 'atypical anorexia nervosa' OR 'anorexia' OR 'anorexia nervosa' OR 'avoidant restrictive food intake disorder' OR 'bulimia' OR 'binge eating disorder' OR 'binge eating' OR 'eating disorder'/de OR 'feeding disorder'/de) AND [2015-2020]/p

**S1.3 – Full Search Terms for LILACS database**

(adolescent OR teen OR child OR youth OR young person OR young people) AND (disordered eating OR Binge-eating disorder OR atypical bulimia OR bulimia OR Anorexia nervosa OR anorexia OR Atypical anorexia OR eating disorders )

**S1.4 – Full Search Terms for PsychInfo**

((((Any Field: (neophobia)))) *OR* (((Any Field: (disordered eating)))) *OR* (((Any Field: (Binge-eating disorder)))) *OR* (((Any Field: (atypical bulimia)))) *OR* (((Any Field: (Bulimia)))) *OR* (((Any Field: (bulimia nervosa)))) *OR* (((Any Field: (ARFID)))) *OR* (((Any Field: (Anorexia nervosa)))) *OR* (((Any Field: (Anorexia)))) *OR* (((Any Field: (Atypical anorexia)))) *OR* (((Any Field: (eating disorders)))) *OR* (((Any Field: (feeding)) *AND* (Any Field: (eating disorders of infancy)) *OR* (Any Field: (early childhood)))) *OR* (((Any Field: (feeding)) *AND* (Any Field: (eating disorders of childhood))))) *AND* ((((Any Field: (epidemiologic methods)))) *OR* (((Any Field: (Probability Sample)))) *OR* (((Any Field: (epidemiolog*)))) *OR* (((Any Field: (incidence)))) *OR* (((Any Field: (cross-sectional studies)))) *OR* (((Any Field: (prevalence)))) *OR* (((Any Field: (prevalence studies)))) *OR* (((Any Field: (prevalence study))))) *AND* ((((Any Field: (adolescent*)))) *OR* (((Any Field: (teen*)))) *OR* (((Any Field: (child*)))) *OR* (((Any Field: (youth)))) *OR* (((Any Field: (young person)))) *OR* (((Any Field: (young people))))) *AND* Publication Type: Peer Reviewed Journal *AND* Year: 2013 *To* 2024

**Table S2 – Data extraction characteristics**

| ***Study characteristics*:** |
| --- |
| - Study location, including country and geographic region - Year of data collection |
| ***Sample characteristics*:** |
| - Sample size - Gender distribution - Age range - Median age - Population type: coded as rural, urban or mixed |
| ***Study methods:*** |
| - Sampling frame, coded as (I) schools, (II) households or (III) population census - Study design, coded as (I) one-stage or (II) two-stage - Sample representativeness, coded as (I) small-medium city or sample, probably representative sample, complex sampling considered; (II) small-medium city or sample, probably nonrepresentative sample or (III) country, weighted to represent the population - Informant, coded as (I) child/young person or (II) child/young person + parent |
| ***Case definition***: |
| - Diagnostic criteria, coded as (I) DSM-5, ( II) ICD-10 or (III) ICD-11 - Diagnostic interview, coded as (I) Kiddie Schedule for Affective Disorders and Schizophrenia (K-SADS), (II) Eating Disorder Examination Questionnaire (EDE-Q), (III) Composite International Diagnostic Interview, (IV) Development and Well-Being Assessment (DAWBA), (V) WHO-CIDI, (VI) Structured Clinical Interview for DSM Disorders or (VII) other clinical interview |
| ***Prevalence data:***   - For the overall/any ED pooled prevalence estimates, each study contributed with one estimate. - If multiple estimates were reported, we selected a single estimate that best reflected our targeted age range (up to 24 years). - When available, prevalence estimates for specific EDs (anorexia nervosa, bulimia nervosa, BED and OSFED) were also extracted and meta-analysed. |

**Table S3 - Quality assessment checklist for prevalence studies (Hoy et al)**

| **Item** |  |  |  |
| --- | --- | --- | --- |
|  |  |  |  |
| **Internal Validity** |  |  |  |
| 1. Was the study's target population a close representation of the national population in relation to relevant variables? **Yes (LOW RISK) or No (HIGH RISK)** |  |  |  |
| 2. Was the sampling frame a true or close representation of the target population? **Yes (LOW RISK) or No (HIGH RISK)** |  |  |  |
| 3. Was some form of random selection used to select the sample, OR was a census undertaken? **Yes (LOW RISK) or No (HIGH RISK)** |  |  |  |
| 4. Was the likelihood of nonresponse bias minimal? **Yes (LOW RISK) or No (HIGH RISK)** |  |  |  |
| **External Validity** |  |  |  |
| 5. Were data collected directly from the subjects (as opposed to a proxy)? **Yes (LOW RISK) or No (HIGH RISK)** |  |  |  |
| 6. Was an acceptable case definition used in the study? **Yes (LOW RISK) or No (HIGH RISK)** |  |  |  |
| 7. Was the study instrument that measured the parameter of interest shown to have validity and reliability? **Yes (LOW RISK) or No (HIGH RISK)** |  |  |  |
| 8. Was the same mode of data collection used for all subjects? **Yes (LOW RISK) or No (HIGH RISK)** |  |  |  |
| 9. Was the length of the shortest prevalence period for the parameter of interest appropriate? **Yes (LOW RISK) or No (HIGH RISK)** |  |  |  |
| 10. Were the numerator(s) and denominator(s) for the parameter of interest appropriate? **Yes (LOW RISK) or No (HIGH RISK)** |  |  |  |
| 11. Summary item on the overall risk of study bias - **LOW RISK 0-3 MODERATE RISK 4-6 HIGH RISK 7-9** |  |  |  |

**Table S4**

**S4 – Meta Regression Results**

**Table S4.1 - Univariate Meta Regression Analysis for Any Eating Disorder (k = 9; n = 50035)**

| **Univariate meta-regression analysis** | | | | | |
| --- | --- | --- | --- | --- | --- |
|  | Estimate | SE | *p* | Lower CI (95%) | Upper CI (95%) |
|  |  |  |  |  |  |
| **Geographic Location** |  |  |  |  |  |
|  |  |  |  |  |  |
| North America (intercept) | 0.0254 | 0.0212 | 0.2301 | -0.0161 | 0.0670 |
|  |  |  |  |  |  |
| Europe | 0.0202 | 0.0261 | 0.4381 | -0.0309 | 0.0713 |
|  |  |  |  |  |  |
| Middle East | -0.0202 | 0.0299 | 0.4999 | -0.0789 | 0.0385 |
|  |  |  |  |  |  |
| Oceania | 0.1966 | 0.0371 | <.0001 | 0.1239 | 0.2692 |
| **Year of Data Collection** |  |  |  |  |  |
| Year as a continuous variable | 0.0070 | 0.0057 | 0.2162 | -0.0041 | 0.0182 |
| **Sample Representativeness** |  |  |  |  |  |
| Small-medium city or sample, probably representative sample  (intercept) | 0.1097 | 0.0505 | 0.0297 | 0.0108 | 0.2086 |
|  |  |  |  |  |  |
| Small-medium city or sample, nonrepresentative sample | 0.0195 | 0.0319 | 0.5414 | -0.0430 | 0.0820 |
|  |  |  |  |  |  |
| Country, weighted to represent the population | 0.0187 | 0.0422 | 0.6580 | -0.0640 | 0.1014 |
| **Study Design** |  |  |  |  |  |
| One-stage (intercept) | 0.0590 | 0.0309 | 0.0559 | -0.0015 | 0.1195 |
|  |  |  |  |  |  |
| Two-stage | -0.0152 | 0.0463 | 0.7428 | -0.1060 | 0.0756 |
| **Diagnostic Interview** |  |  |  |  |  |
| EDDS (intercept) | 0.0370 | 0.0265 | 0.1633 | -0.0150 | 0.0890 |
|  |  |  |  |  |  |
| SIAB-S | -0.0220 | 0.0375 | 0.5576 | -0.0955 | 0.0515 |
|  |  |  |  |  |  |
| KSADS | -0.0260 | 0.0324 | 0.4225 | -0.0896 | 0.0375 |
|  |  |  |  |  |  |
| DAWBA | 0.0261 | 0.0327 | 0.4245 | -0.0380 | 0.0903 |
|  |  |  |  |  |  |
| EDE-Q | 0.1850 | 0.0378 | <.0001 | 0.1109 | 0.2591 |
|  |  |  |  |  |  |
| EDE | 0.0041 | 0.0378 | 0.9135 | -0.0699 | 0.0781 |
|  |  |  |  |  |  |
| SCID | -0.0345 | 0.0374 | 0.3562 | -0.1078 | 0.0388 |
| **Sampling Frame** |  |  |  |  |  |
| School (intercept) | 0.0168 | 0.0495 | 0.7337 | -0.0801 | 0.1138 |
|  |  |  |  |  |  |
| Household | 0.0465 | 0.0391 | 0.2345 | -0.0302 | 0.1232 |
|  |  |  |  |  |  |
| Multiple frames | -0.0325 | 0.0781 | 0.6771 | -0.1856 | 0.1205 |
| **Diagnostic Criteria** |  |  |  |  |  |
| DSM-IV-TR (intercept) | -0.0373 | 0.0564 | 0.5092 | -0.1479 | 0.0734 |
|  |  |  |  |  |  |
| DSM-5 | 0.0590 | 0.0302 | 0.0505 | -0.0001 | 0.1181 |
|  |  |  |  |  |  |
| ICD-10 | 0.0070 | 0.0566 | 0.9020 | -0.1039 | 0.1178 |
| **Informant** |  |  |  |  |  |
| Child/young person (intercept) | 0.0634 | 0.0306 | 0.0381 | 0.0035 | 0.1232 |
|  |  |  |  |  |  |
| Child/young person + parent | -0.0250 | 0.0458 | 0.5856 | -0.1149 | 0.0649 |
| **Prevalence timeframe** | | | | | |
| 0.5 month (intercept) | 0.0110 | 0.0380 | 0.7724 | -0.0635 | 0.0855 |
|  | | | | | |
| 1 month | 0.1205 | 0.0539 | 0.0254 | 0.0148 | 0.2262 |
|  | | | | | |
| 3 months | 0.0260 | 0.0450 | 0.5636 | -0.0623 | 0.1143 |
